# Altruistic punishment is connected to trait anger, not trait altruism, if compensation is available

**DOI:** 10.1016/j.heliyon.2018.e00962

**Published:** 2018-11-27

**Authors:** Johannes Rodrigues, Natalie Nagowski, Patrick Mussel, Johannes Hewig

**Affiliations:** a Julius-Maximilians-Universität Würzburg, Marcusstraße 9-11, 97070, Würzburg, Germany; b Universität Osnabrück, Seminarstr. 20, 49074, Osnabrück, Germany; c Freie Universität Berlin, Habelschwerdter Allee 45, 14195, Berlin, Germany

**Keywords:** Psychology

## Abstract

Altruistic punishment and altruistic compensation are important concepts that are used to investigate altruism. However, altruistic punishment has been found to be correlated with anger. We were interested whether altruistic punishment and altruistic compensation are both driven by trait altruism and trait anger or whether the influence of those two traits is more specific to one of the behavioral options. We found that if the participants were able to apply altruistic compensation and altruistic punishment together in one paradigm, trait anger only predicts altruistic punishment and trait altruism only predicts altruistic compensation. Interestingly, these relations are disguised in classical altruistic punishment and altruistic compensation paradigms where participants can either only punish or compensate. Hence altruistic punishment and altruistic compensation paradigms should be merged together if one is interested in trait altruism without the confounding influence of trait anger.

## 1 Introduction

Altruistic behavior has been defined as a voluntary action intended to benefit another person without the expectation of receiving external rewards or avoiding externally produced aversive stimuli or punishments (Eisenberg and Miller, 1987). However, this broad motive definition of altruism has been further narrowed down in a factor-analytical conceptualization (Carlo et al., 2003; Carlo and Randall, 2002) where prosocial behavior has been categorized according to their driving motives into six different categories (see also Rodrigues et al., 2017 for validation in German). Among these six categories was altruism, defined by Carlo and Randall (2002), seeing altruism or altruistic prosocial behavior as “voluntary helping motivated primarily by concern for the needs and welfare of another, often induced by sympathy responding and internalized norms/principles consistent with helping others” (Carlo et al., 2010, p. 273). Also, as the helper is more concerned about the need of the others, costs that may occur are included in the definition as well (Carlo and Randall, 2002). Accordingly, we use altruism as defined by Carlo and Randall (2002) as a trait-construct that is motivationally based on the concern for the needs and welfare of others and related to affective reactions like empathy and sympathy (Eisenberg et al., 2014). In context of economic decision making games, behavioral altruism has been defined in a similar way as costly action of benefit to another person (Fehr and Fischbacher, 2003). Some of the behavioral paradigms that are used to assess altruistic behavior (see e.g. Fehr and Gächter, 2002) include third party dictator games (Leliveld et al., 2012; Lotz et al., 2011b; Strobel et al., 2011). In this variant the participants assume the role of an observer, watching interactions or outcomes of interactions between a dictator and a responder in a dictator game. The dictator divides a given amount of money between himself and the responder. As usual in dictator games the responder cannot act and simply receives the allotted amount. The observer on the other hand is endowed with his own fixed amount of money. Following the dictators allocation, the observers may act themselves. Their scope of actions varies from study to study. In studies investigating altruistic punishment the observer has the opportunity to use his money to punish the dictator i.e. decreasing the money of the proposer. Another version of the third party dictator game gives the observers the chance to compensate i.e. increase the money of the responder or choose between compensating the responder and punishing the dictator (Leliveld et al., 2012).

### 1.1 Altruistic punishment

A question arising in this context is whether altruistic punishment is actually altruistic by its nature. While punishment is a costly behavior it has no obvious direct benefits for another person. Based on the aforementioned definition this behavior is not altruistic in a more narrow sense. However, it has been argued that altruistic punishment has indirect altruistic side effects by increasing conformity to social norms that increase cooperation (Fehr and Gächter, 2002). Additionally, it was found recently, that people in dictator games are prone to show behavior that is framed as morally right (Capraro and Rand, 2018; Tappin and Capraro, 2018), which might also lead to altruistic punishment in third party economic games if altruistic punishment is the only available behavioral option. Fehr and Gächter (2002) showed that situations leading to altruistic punishment also evoke negative emotions like anger and Jordan et al. (2016) showed, that altruistic punishment is related to subjective ratings of state anger. Also, the offender focused emotion rating of Lotz and colleagues (2011b) showed a strong relation of anger towards the offender with altruistic punishment. Additionally, anger has been found to be a mediator of altruistic punishment (Seip et al., 2009), being a better predictor to altruistic punishment than perceived unfairness (Pillutla and Murnighan, 1996). The concept of anger is defined as “the response to interference with our pursuit of a goal we care about. Anger can also be triggered by someone attempting to harm us (physically or psychologically) or someone we care about. In addition to removing the obstacle or stopping the harm, anger often involves the wish to hurt the target” (Ekman and Cordaro, 2011, p. 365). Acting out anger should also not be seen as destructive and negative act per se, but this acting out of anger can also be used in a constructive manner (Ekman and Cordaro, 2011), like punishing defectors in dictator games to cause a possible change in their behavior in future trials (Leliveld et al., 2012). But not only state anger might be relevant for altruistic punishment. Individual differences in the proneness to experience anger have been investigated for a long time (e.g. Spielberger, 1988) and the construct of trait anger has been found to be related to behavior like the approach of hostile situations (Veenstra et al., 2017) and cyberbullying (Lonigro et al., 2015). As trait anger is also linked to higher aggression (Veenstra et al., 2018), the link between trait anger and acting out a punishment as an reaction to the norm violation may be given. Further studies have identified and corroborated several correlates of altruistic punishment like guilt and anger (Nelissen and Zeelenberg, 2009) as well as altruism (Strobel et al., 2011) and envy (Pedersen et al., 2013). Therefore different kinds of motives may be hidden behind third party punishment behavior, but anger plays an important role to get the punishment going (Seip et al., 2009).

### 1.2 Traits as capabilities to react to state manipulations

As the focus of research on this topic used to be on the state component of anger, we tried to investigate trait anger and its influence on “altruistic” punishment. Using this trait approach, one may be able to explain trait based variance in state based changes, in order to come to a more precise prediction. For example, a person with low trait anger scores could not get angry during a high state anger induction paradigm, while a high trait anger person might react quicker and more easily to this anger inducing procedure. These “capabilities” to react to a certain state manipulation based on personality traits are known in many research fields, for example for physiological reactions like frontal asymmetry (see capability model, Coan et al., 2006). More general, one can also consider the latent state-trait model (Steyer et al., 1999) as a different variant to include state and trait variance in order to explain the behavior. Hence, we also wanted to investigate the trait effects of anger on altruistic punishment to achieve an estimation of possible systematic error variances that may be considered when analyzing only state anger.

### 1.3 Altruistic compensation

Third party compensation on the other hand is more likely to be primarily driven by the latter more narrow altruistic motivation defined by Carlo and colleagues (2002, 2003) and has been found to be related with empathic concern (Leliveld et al., 2012). To illustrate this idea of altruistic compensation in contrast to altruistic punishment, a short hypothetical example is given. If one thinks about a man being pushed to the ground, a person with high trait anger will primarily react with anger towards the aggressor, whereas a person with high trait altruism will primarily react with empathy for the victim. Altruistic compensation does not accept a possible harm either for the aggressor or the victim, and therefore it is in accord with the definition of altruistically motivated behavior because the welfare of the persons, even the welfare of the possible aggressor is not endangered (see Carlo et al., 2010, Eisenberg et al., 2007), in contrast to altruistic punishment.

### 1.4 A narrow definition of altruism

Following this example, we even suggest to explicitly include this “benevolence” in a narrow definition of altruism. Altruism and altruistic acts following our view and extending the work of Carlo and colleagues (2010), would be an action that is voluntary, intended to benefit another person, driven by this motivation to help the other person to at least 50% (in order to avoid the domination of other motives like public reputation, see e.g. Carlo and Randall, 2002; Rodrigues et al., 2017) and is benevolent, meaning that there is no intention of harming other persons during the process of helping. This narrow definition of altruism is an extension of the definition given by Carlo and colleagues (2010) and is in contrast to the definition given by Fehr and Fischbacher (2003), which only includes the costs of the action and the benefit for another person. In the case of “altruistic punishment”, this benefit is argued to be given by the reinforcement of a fairness norm, but we would doubt that the driving force behind this action is altruism. Instead, we would suggest that trait anger might play a more important role for “altruistic” or maybe more precisely “costly” punishment.

### 1.5 Hypotheses

Given the empirical evidence and our definition of altruism mentioned above, we aimed to examine the prevailing motivation in third party punishment as compared to third party compensation using individual differences in trait altruism and trait anger. Although these two concepts are different kinds of traits, for trait anger being the proneness to experience a basic emotion (Ekman and Cordaro, 2011) and altruism being a facet of prosocial behavioral tendencies (Carlo and Randall, 2002), they were chosen because of their empirical relation to the behavioral options altruistic punishment and altruistic compensation. Also, they are both seen as facets of the big five personality traits, with anger being the second facet of neuroticism and altruism being the third facet of agreeableness (Maples et al., 2014). Therefore, these two yet different traits may be comparable concerning their effects on third party paradigms. We hypothesized that altruistic punishment would correlate positively with measures of trait anger and aggression whereas altruistic compensation would correlate positively with a measure of trait altruism. To control for the potential influences of the behavioral options provided by the paradigm used to study altruism, in this case providing only the option to punish or only the option to compensate and therefore measuring a combination of altruism and anger, we included three different blocks in the experiment, where the observer could only punish, only compensate or do both.

## 2 Materials and methods

### 2.1 Ethical statement

The study was carried out in accordance with the recommendations of “Ethical guidelines, The Association of German Professional Psychologists” (“Berufsethische Richtlinien, Berufsverband Deutscher Psychologinnen und Psychologen”) with written informed consent from all subjects. All subjects gave written informed consent in accordance with the Declaration of Helsinki before they participated in the experiment. The protocol was not approved by any additional ethics committee, for the used paradigms are common practice in psychological experiments. Also, following §7.3.2 of the “Ethical guidelines, The Association of German Professional Psychologists”, the approval by an ethical committee is optional. As the local ethics committee is very busy, it does not deal with paradigms that are common practice and ethically uncritical. The local ethics committee only handles potentially problematic experiments and as all ethical standards and recommendations were complied, and the study protocol was deemed uncritical concerning ethical considerations, the study was not submitted to the local ethics committee. Additionally, researchers have the responsibility for conducting their research according to the human rights and ethical guidelines, independent of being approved by an ethic committee or not. An ethic committee approval does not change the responsibility of the researcher. Accordingly, the study did not receive and does not require an ethical committee approval according to our institution's guidelines and national regulations. During the experiment, a cover story was used, but they were told about this deception as soon as the task was over, as it is common practice in psychological experiments.

### 2.2 Participants

We a priori estimated the required sample size with G-power software (Faul et al., 2007). Assuming an average effect of r = .36 of anger on altruistic punishment (e.g. Lotz et al., 2011a) and α = .05 and power (1-β) = .8 yielded a required sample size of N = 55. 58 participants participated in this study to account for possible data loss. Missing data occurred eventually for one person, because the number of the online questionnaires were lost and leading to a final sample size of 57 participants (29 females, 28 males, mean age = 23.11, SD age = 6.91, range = 18–52, 4 left handed). Despite all participants having the illusion that they would get money from the experiment because of the cover story, most of the participants received educational credits for their participation, the rest of the participants were paid a small amount of money (5 Euro) for their participation.

### 2.3 Procedure

Participants were told that they were part of a cooperation based study with other universities, investigating economic decisions under time pressure. This setting was used as a cover story and was not revealed to the participants until the end of the experiment in order to convince the participants that they were playing with other persons in the third party economic game. Also, as the other fictive players were from other universities and not a direct “ingroup”, the cooperation was not directly reinforced. First they filled in a web-based questionnaire, containing several trait questionnaires (see trait measurement section) and demographical data (e.g. gender, age and handedness). The online questionnaire was presented with SoSci Survey (Leiner, 2014).

Then the participants came to the lab for the experiment. They were told that three different roles were provided in this study which would be randomly assigned: The first role would be the dictator who has to divide 8 Cents between him- or herself and a receiver, the second position. The third position would be a spectator of the dictator's offer. The player in the third position would be able to interfere with the resulting division by investing his or her own money. Unbeknownst to the participants, the lottery assigning positions was staged so that participants always participated in the role of the spectator. Because they were the only person actually participating in the study, the other positions were played by the computer, which was not revealed to the participants until the end of the experiment.

The experiment was divided into three blocks: In the first block, participants were only able to punish the fictive dictator by spending their money. The participants were told that for each cent spent, the dictator lost one cent. No additional information was given and no additional framing was intended. This is the classical third party punishment paradigm or altruistic punishment game as used by Fehr and Fischbacher (2004). In the second block the participants could only compensate the receiver with their money. For each cent spent the receiver got an additional cent. No additional information was given and no additional framing was intended. This is the altruistic compensation game as used by Leliveld et al. (2008). In the third block the participants could either punish the dictator as in the first block or compensate the receiver as in the second block or do both. First they were able to punish the dictator as in the first block followed by the opportunity to compensate the receiver. At the end of the trial the resulting allocation was shown as in block 1 or block 2. In this third block the participants were able to spend twice as much as in the first and second block. But the maximum and minimum of the resulting amounts of money for dictator and receiver always stayed between the same boundaries as in the previous blocks (for a more detailed example see below) and the ratio between money spent by the dictator and the money the participant is able to spend in every part of the task stayed the same. Each of the three blocks consisted of 45 trials and the participants were informed of their type of interaction just before the block started. So they had no knowledge during a block what kind of interaction with the other fictive players would occur in the next blocks. Also, the participants were not informed at the beginning of the experiment what kind of interaction exactly would be possible during the experiment. Hence, the participants had just the information what to do in the present block. All 45 offers were randomly sampled from the three offers (offering 0 cent, 2 cent or 4 cent) in the offer range from 0 to 8 cents as explained below, with each offer being presented 15 times. All trials started with the alleged offer of a fictive dictator shown for 1.5 seconds depicted as picture of an offer with either 8:0, 6:2 or 4:4 cents and therefore always leaving at least one half of the money for the fictive dictators. Then participants had the opportunity to spend their money for 5 seconds. This time constraint was imposed because of the cover story and to keep the experiment time under control, for the free choice time could lead to very long trials. The amount of money participants were able to spend was identical to the money kept by the dictator. For example if dictators kept 8 Cents for themselves a maximum of 8 Cents could be spent. So a participant could use all available money for punishment and the dictator would get 0 cent. Thus the resulting amount of money for dictator and receiver were kept between the same boundaries. We only analyzed the relative amount of money spent, meaning the amount of money that was spent by the participant, divided by the amount of money that was available to spent in the respective trial, in order to correct for the different reference frames of the meaning of e.g. 2 Cents when one has 6 Cents to spent vs. 2 Cents to spent. After making a decision or after 5 seconds had passed the trial continued with showing the resulting allocation for the three parties for1 second. Thereafter, a fixation cross was shown for 3 seconds, keeping up the cover story of sending and receiving the data from the other participants before the next trial started. The first two blocks were followed by a break of 15 seconds each.

It was not stated clearly to the participants whether the other (fictive) players would be the same for the whole game or not, but the setting of the cover story suggested that they would be playing with the same persons during the whole experiment.

### 2.4 Trait measurement

The questionnaires used in this study were a translated version of the revised version of the Prosocial Tendencies Measure (PTM-R; Carlo and Randall, 2002; Carlo et al., 2003, Rodrigues et al., 2017), the German version of Buss – Perry aggression questionnaire (Buss and Perry, 1992; Herzberg, 2003), the German version of State- trait – anger – expression – inventory (STAXI; Schwenkmezger and Hodapp, 1991; Spielberger, 1988) and a German version of the empathic concern scale (Paulus, 2009).

For the PTM-R, the subscale altruism was used to determine altruism on a trait level (*Cronbach's α* = .67). This scale consists of 6 items, like the negatively poled item “*I think that one of the best things about helping others is that it makes me look good*”.

For the Buss – Perry aggression questionnaire, the subscale anger (*Cronbach's α* = .82) was used to determine anger on a trait level, along with the measurement of STAXI (*Cronbach's α* = .77). This scale consisted of 7 items, like the positively poled item *“I sometimes feel like a powder keg ready to explode”*.

The two different measurements of anger were not averaged, as the STAXI was used to assess the trait anger explicitly, while the Buss-Perry aggression questionnaire did not distinguish that clearly between state and trait anger. Therefore the measurements obtained with the Buss-Perry aggression questionnaire were only included in the exploratory analysis.

For the exploratory analysis, we used the subscales of aggression (measured with Buss - Perry aggression questionnaire Herzberg, 2003, *Cronbach's α* = .89) and empathy (measured with empathic concern scales Paulus, 2009, *Cronbach's α* = .46).

### 2.5 Data analytic approach

We computed four linear regressions with the mean of the relative amount of money spent in every condition (“punishment only”, “compensation only”, “punishment if both options are available”, “compensation if both options are available”) as the criterion for each of two predictors: “Trait altruism” (measured with PTM-R; Carlo et al., 2003) and “trait anger” (measured with STAXI; Schwenkmezger and Hodapp, 1991). Following our hypothesis, we expected trait altruism to predict compensation and trait anger to predict punishment. Additionally, we made two linear regressions for the third block of the experiment were both behavioral options were available with the “trait altruism”/“trait anger” as criterion and the mean relative amount of money used for “punishment” and “compensation” as predictors.

In addition we exploratory analyzed the correlations between the subscales of aggression (measured with Buss - Perry aggression questionnaire Herzberg, 2003) and empathy (measured with empathic concern scales Paulus, 2009) with the amount of money spent in all conditions.

Statistical analysis was carried out with IBM SPSS version 21.

The data analyzed in this study is provided as supplementary data in order to be available for meta-analyses or re-analyses.

## 3 Results

The reliability of all questionnaire scales included in the analyses is shown in Table 1.

**Table 1:** Cronbach's alpha for every questionnaire included in the analyses.

Scale	PTM-R altruism	STAXI trait anger	Buss - Perry aggression questionnaire aggression	Buss - Perry aggression questionnaire anger	Buss - Perry aggression questionnaire physical aggression	Buss - Perry aggression questionnaire verbal aggression	Buss - Perry aggression questionnaire hostility	SPF empathic concern
Cronbach's alpha	0.67	0.77	0.89	0.82	0.72	0.64	0.77	0.46
Number of items	6	10	27	6	8	5	8	4

The mean relative money spent on compensation and punishment in every block for every offer of the dictator can be seen in Table 2.

**Table 2:** Mean relative and mean absolute money spent for every behavioral condition of the experiment and for every offer.

	Whole experiment	Offer of dictator: 0 cent	Offer of dictator: 2 cent	Offer of dictator: 4 cent
Mean relative money used for punishment only	36%	49%	39%	19%
Mean relative money used for compensation only	29%	41%	33%	13%
Mean realtive money used for punishment if both was available	28%	38%	29%	18%
Mean realtive money used for compensation if both was available	39%	46%	41%	29%

Mean absolute money used for punishment only in cents	2.33	3.89	2.35	0.76
Mean absolute money used for compensation only in cents	1.92	3.28	1.97	0.51
Mean absolute money used for punishment if both was available in cents	1.84	3.03	1.77	0.71
Mean absolute money used for compensation if both was available in cents	2.45	3.70	2.47	1.17

For the regression models with the traits as predictors, only two regression models of the first 4 regression models show a significant effect of the predictor and one regression model shows a marginal effect for the predictor on the behavior. Summaries of these regression models are shown in Table 3. For “altruism” as a predictor for the criterion “compensation if both options are available” *β* = .297, *t(55)=*2.15, *p <* .05, for “anger” as predictor for the criterion “punishment if both options are available” *β* = .249, *t(55)=*1.79, *p=*.08 and for “anger” as predictor for the criterion “punishment only” *β* = .299, *t(55)=*2.16, *p <* .05 significant effects were found. All in all, the regression analyses showed that if participants have the option to either punish or compensate, then people scoring high on anger are more likely to punish whereas high altruists are more likely to compensate.

**Table 3:** Summary of regression models.

Predictor	Criterion	Significant	*t*	*p*	*β*	*R*^*2*^
Altruism	Punishment only	−	1.50	.138	.208	.09
Anger	Punishment only	+	2.16	.035	.299	.09
Altruism	Compensation only	−	1.56	.124	.221	.05
Anger	Compensation only	−	.89	.377	.126	.05
Altruism	Punishment, given both options	−	−0.37	.710	−.052	.04
Anger	Punishment, given both options	(+)	1.79	.080	.249	.04
Altruism	Compensation, given both options	+	2.15	.036	.297	.08
Anger	Compensation, given both options	−	0.19	.849	.027	.08
Punishment, given both options	Altruism	(+)	−1.78	.081	−.235	.13
Compensation, given both options	Altruism	+	2.68	.01	.352	.13
Punishment, given both options	Aanger	+	2.31	.025	.311	.10
Compensation, given both options	Anger	−	−1.19	.238	−.160	.10

The two regression models with the traits as criterion for the third block revealed that persons showing more compensation in this block had higher altruism scores *β* = .352, *t(54)=*2.68, *p <* .05. Also, participants that showed more punishment were marginally significantly less altruistic *β* = −.235, *t(54)=*−1.78, *p=*.08 and had significantly higher anger scores *β* = .311, *t(54)=*2.31, *p <* .05.

**Fig. 1:**
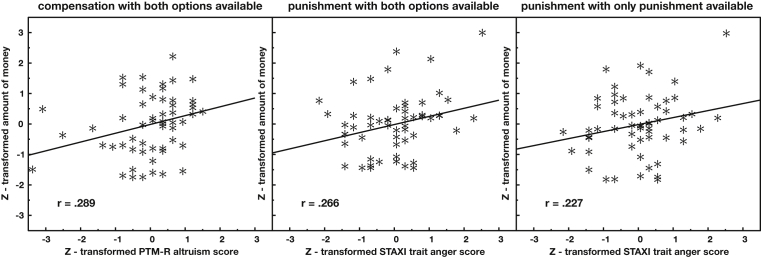
Correlation of trait altruism and trait anger with the relative amount of money spent for altruistic compensation or altruistic punishment.

The bivariate correlations of the relevant parameters in the significant and marginally significant regression models are shown in Fig. 1.

Exploratory analysis revealed significant correlations between the mean of the relative amount of money spent in the different conditions as can be seen in Table 4. The subscales hostility and verbal aggression from Buss – Perry Aggression Questionnaire (Herzberg, 2003) show a marginally significant correlation (hostility: *r* = .234, *p* = .079, verbal aggression: *r* = .242, *p* = .070) with the amount of money spent in the punishment only condition. Other personality traits than those that were already tested with regression models do not show a significant correlation with the amount spent on punishment or compensation. Possible income effects, leading to less investment if one has invested much in prior blocks can be ruled out by the positive correlation of all behaviors in all blocks.

**Table 4:** Correlation of money spent in altruistic punishment, money spent in altruistic compensation, empathic concern scales and Buss – Perry Aggression Questionnaire with subscales hostility, physical aggression, verbal aggression and anger.

	Punishment only	Compensation only	Punishment given both options	Compensation given both options
Compensation only	.624**			
Punishment given both options	.532**	.565**		
Compensation given both options	.642**	.661**	.287*	
Aggression	.197	.033	.079	−.098
Hostility	.234^†^	.076	.142	−.044
Physical aggression	−.012	−.06	.045	−.123
Verbal aggression	.242^†^	.113	−.011	−.003
Anger	.131	−.029	.056	−.137
Empathic concern	−.027	.011	−.131	.039

Notes: **: p < .01, *: p < .05, †: p < .1.

## 4 Discussion

Besides using helping behavior as well as dictator games, third party dictator games were also used to account for altruistic behavior (see Fehr and Gächter, 2002). In these third party dictator games the two major varieties that were used are altruistic punishment and altruistic compensation. Our study investigated whether altruism is the driving motivation for altruistic punishment and compensation or whether anger plays the major role in altruistic punishment.

### 4.1 Trait anger leads to punishment, trait altruism leads to compensation

We found that given both opportunities to punish and to compensate, the relative amount of money spent in the task is predicted by trait anger in the case of altruistic punishment and by trait altruism in the case of altruistic compensation. Thus altruistic punishment seems to be driven more by trait anger than by trait altruism, if both options are available. Also, trait altruism does not predict altruistic punishment if both behavioral options are given. We could also show that this effect is true in general, not just in the case given both options. We also found an effect of the availability of just one option vs the two behavioral options. Here, just having the option to punish leads to more punishment compared to the punishment that is given if both options are available. Remarkably there was no significant interaction of the possibility to punish or to do both punishment and compensation, with trait anger in predicting the altruistic punishment. The opposite pattern can be observed for altruistic compensation, where trait altruism seems to be the driving force of the shown behavior, also with an additional effect of the behavioral options, where having both options leads to more compensation but still there is no significant interaction of these two effects.

### 4.2 The importance of having both behavioral options available and executed

However, as there is a high correlation between the assessed behaviors in the different tasks, there still might be a conglomeration of trait anger and trait altruism driving the resulting behavior. Therefore it is not possible to get an uncontaminated measure of one or another if one just uses one behavioral option, either to compensate or to punish the other players in the third party economic game. But if there are two possible options, the option to punish the dictator and the possibility to compensate the receiver, the influence of altruism on the altruistic compensation and the influence of anger on the altruistic punishment are strengthened and the other trait loses influence on the behavior. Hence it is important to use a task that combines both paradigms, altruistic punishment and altruistic compensation instead of using just one behavioral option, if one is interested in the measurement and influence of altruism and anger on these economic decisions. Leliveld and colleagues (2012) as well as Lotz and colleagues (2011b) did already make that notion on another behalf, showing that it is important to give participants the opportunity to choose what kind of behavior they want to execute. Furthermore they could show the influence of empathic concern (Leliveld et al., 2012) and offender focused emotion (Lotz et al., 2011b) on the choice of compensating or punishing behavior in third party economic games. Our work is trying to extend these findings to the motivational level, now showing that the narrow altruistic motive is not linked to the punishing behavior.

### 4.3 Implications of the findings for society

Therefore, the altruistic consequences of the punishment behavior might not be the primary concern of the actor, but just the mere thought of retribution or reinforcement of social norms (see Leliveld et al., 2012, Lotz et al., 2011a). As long as the punishment stays in between boundaries of adequacy, this may be a good way to strengthen the social norms in a society, but this kind of behavior might actually damage a society if one punishes to hard. Furthermore, the immediate problem of the receiver, in this case having less or no money at all, is not targeted by this kind of behavior, so the decline of the welfare of this person is accepted and a purely altruistic motive is therefore unlikely. The act of compensation on the other hand is linked to altruistic motivation, targeting the welfare of the receiver right away, but ignoring a possible perseverance of unfair behavior in the society. Thus altruistic compensation, besides being linked to altruistic motivation and closely related to the definition of altruism (e.g. by Eisenberg et al., 2007), just leads to a short sided welfare effect for the person supposedly in need, but does not have the intend to change the behavior or even harm or punish defectors of social norms and the society.

### 4.4 Trait anger and trait altruism in the third party economic game with both behavioral options

In this study, we used trait altruism and trait anger as predictors for punishment behavior as well as for altruistic compensation. One reason to do so was to account for the problem of unexplained variance that may occur if one is only dealing with induced states in such a paradigm (see e.g. Steyer et al., 1999). Traits like anger and altruism may act as a heuristic for reactions in people (e.g. Veenstra et al., 2017, 2018; Rodrigues et al., 2015). Hence traits may in some cases overshadow state manipulations that are given and therefore lead to systematic error variance, if only the states are considered as relevant. Another reason to include stable dispositions of people in this experiment was to investigate the reaction patterns related to relevant traits if different behavioral options are given. As trait anger was always related to punishment behavior, the behavioral options do not seem to have an impact on people with high trait anger. They will likely try to punish defectors, even if they have the additional chance to help the victims of the defection. This kind of behavior is not to be seen as a bad thing per se, as long as the punishment stays in appropriate boundaries. Some advantages and disadvantages of the punishment behavior and altruistic compensation for the individual and the society have been shortly mentioned above, and the purely altruistic act of compensation is not likely to cause a change in the behavior of the defector and his impact on society. If only the option to compensate is present, there is no specific reaction pattern for high or low trait anger. Hence they will just help as everyone else would do with no specific deviation from it. Trait altruism however only shows a clear influence if one is able to help the victim and to punish the defector. A specific negative relation to punishment was present and a positive relation on helping the victim was found. But if punishment or compensation was the only behavioral option, no specific relation was found. Therefore high trait altruism people as well as low altruism people will also go for the punishment like everyone else, if they don't have any other option to react. These findings lead to the assumption, that if one only provides the behavioral option of helping, everyone may choose this option, independent of their trait disposition, as long as a motivation to show any reaction is present. For punishing behavior on the other hand, trait anger seems to always play an important role. This leads to simple practical implications concerning behavioral options and confounds of trait motivation that could be used in our society. As long as one is only providing benevolent behavioral options, everyone may choose them in order to satisfy their urge to react according to their traits. But as soon as some other options are available, the traits will take their influence in choosing relevant behavioral options like punishment in the case of trait anger. Therefore every association, society or movement should consider whether they want to engage in purely benevolent actions like for example cleaning the shores in order to get everyone involved in these actions, or whether they want to provide also more punishment prone activities like for example blockading or even attacking an oil platform, which would automatically lead to a division of their members, likely based on traits and motivations like anger and altruism. Also, calling destructive and aggressive acts altruistic may not be the right labeling, for they are most likely driven by anger or trait anger and should therefore not be called altruistic.

### 4.5 Limitations

One limitation of the present work is the confounding of the different options of interaction with the order in the different blocks. As the participants experienced the two blocks with the options of punishment and compensation first, before they learned about their more complex task to do both, they all experiences the option where both behavioral opportunities were present at the end of the paradigm. This order was chosen to make sure that the participants are able to deal with the more complex task on one hand, on the other hand, that they do not feel bored in the blocks after the complex task with the simpler ones. Also in order to not work against the impulsive component of anger, the order of the block during the paradigm was chosen with the punishment always being the first option in block three or being the first behavior to execute in the paradigm in block one.

A second limitation is the time constraint that was implemented for the decision of the participants. This may have an influence on the amount of punishment and compensation that is shown by the participants. Rand (2016) argues that more intuitive driven paradigms, as operationalized with the time constraint in our paradigm, lead to more cooperation. Also, Rand and colleagues (Rand et al., 2016) found that this effect of intuition driven paradigms is true for women, but not for men. However Capraro and Cococcioni (2016) showed that a strong time constraint may also lead to decreased cooperation via ego-depletion. But these finding do not lead to a clear prediction of the bias in the present paradigm, because no third party economic game was included in both studies. One may only guess that the altruistic compensation might be higher under time pressure, for it is more similar to the cooperation behavior that was assessed in the meta-analysis by Rand (2016) than altruistic punishment. However Sutter et al. (2003) could show that a tight time constraint leads to more rejection and therefore altruistic punishment in the ultimatum game, although the effect vanishes with repetition. Therefore, we could also expect an initial higher altruistic punishment in third party games under the time constraint that is implemented here as we would expect without it. But as the bias should influence both altruistic punishment and altruistic compensation in the same manner (see above except Capraro and Cococcioni, 2016), the time constraint should not add systematic error variance to the findings.

Another limitation of the present study is the sample size. However, as the power of the study was estimated, we are confident, that this work might contribute to the field none the less. Also, the reliability of some scales involved in this study was rather low (see Table 1) and this may influence also the reliability of the conclusions drawn from the data.

## 5 Conclusion

Importantly recent studies using compensate only or punishment only paradigms have systematically confounded the motives of trait altruism and trait anger. Our findings are in line with previous results suggesting a strong relation between anger and altruistic punishment (see Fehr and Gächter, 2002; Nelissen and Zeelenberg, 2009). If given the choice, high trait altruists seem to prefer compensation, which is perfectly well in line with the narrower view and definition of altruism being revealed by a voluntary action of benefit to another person without the intention of harming other persons. Accordingly, our results corroborate the view that different kinds of motives and traits may be hidden behind third party punishment behavior and that altruistic punishment is not related to altruism, if an option of compensation is available.

## Declarations

### Author contribution statement

Johannes Rodrigues: Conceived and designed the experiments; Performed the experiments; Analyzed and interpreted the data; Contributed reagents, materials, analysis tools or data; Wrote the paper.

Natalie Nagowski: Conceived and designed the experiments; Performed the experiments; Analyzed and interpreted the data.

Patrick Mussel: Analyzed and interpreted the data.

Johannes Hewig: Conceived and designed the experiments; Analyzed and interpreted the data.

### Funding statement

This work was supported by the German Research Foundation (DFG) and the University of Wuerzburg in the funding programme Open Access Publishing. Also, it was funded by the European Union through the project “Individualisierung Digital” (Fonds 823881) in the Europäischer Fonds für regionale Entwicklung (EFRE).

### Competing interest statement

The authors declare no conflict of interest.

### Additional information

No additional information is available for this paper.

## Appendix A Supplementary data

Supplementary Data.zip Supplementary Data.zip
